# Prevalence of bovine subclinical mastitis and isolation of its major causes in Bishoftu Town, Ethiopia

**DOI:** 10.1186/s13104-017-3100-0

**Published:** 2017-12-21

**Authors:** Misrak Birhanu, Samson Leta, Gezahegne Mamo, Shimelis Tesfaye

**Affiliations:** 1 Faculty of Veterinary Medicine, University of Gondar, P.O. Box 196, Gondar, Ethiopia; 2 College of Veterinary Medicine and Agriculture, Addis Ababa University, P.O.Box 34, Bishoftu, Ethiopia

**Keywords:** Bishoftu, Dairy, Prevalence, Subclinical mastitis

## Abstract

**Objective:**

A cross-sectional study was conducted from November 2015 to March 2016 to estimate the prevalence, to assess the risk factors and to isolate the major etiological agent of subclinical mastitis in Bishoftu town. The study was conducted on 262 cross breed lactating cows selected from 12 intensively managed dairy farms. California mastitis test (CMT) and bacteriological culture methods were used as diagnostic tools.

**Result:**

From 262 cows examined, 105 (40.1%) and from 1048 quarters examined, 170 (16.1%) were positive for sub-clinical mastitis using CMT. All CMT positive samples were cultured for etiological agent identification. From 170 samples cultured, 153 were positive for known subclinical mastitis pathogens. The dominant bacteria isolated were *Staphylococcus* species from these *Staphylococcus aureus* (44.9%) was the major one followed by *Streptococcus* spp. (25.3%) and other gram negative enteric bacteria, *Escherichia coli* (8.8%). Age, body condition score, milk yield, and number of parity were considered as potential risk factors; among these, age and number of parity have statistically significance association with the occurrence of subclinical mastitis (P < 0.05) both in the CMT and the bacteriological tests.

## Introduction

Ethiopia has one of the largest livestock populations in Africa [1–3]. The livestock subsector has an enormous contribution to Ethiopia’s national economy and livelihoods of many Ethiopians, and still promising to rally round the economic development of the country. Livestock plays vital roles in generating income, creating job opportunities, ensuring food security, providing services, contributing to asset, social, cultural and environmental values, and sustain livelihoods [4].

Despite high livestock population and existing favorable environmental conditions, the contribution of the livestock subsector to the Ethiopian economy is below the potential. This is associated with a number of complex and inter-related factors such as inadequate feed and nutrition, widespread diseases, poor genetic potential of local breeds, market problem, and inefficiency of livestock development [2, 5–7]. Mastitis is one of the major and expensive diseases in terms of production losses in dairy production [8, 9]. It severely reduces milk yield, profit margins, and quality of milk and milk products in all dairy producing countries of the world [10].

Mastitis can be classified as either clinical or subclinical. Clinical mastitis is characterized by sudden onset, alterations of milk composition and appearance, decreased milk production, and the presence of the cardinal signs of inflammation in infected mammary quarters. In contrast, in subclinical mastitis no visible signs are seen either on the udder or in the milk, but the milk production decreases and the somatic cell count increases [8]. According to Mungube et al. [11], subclinical mastitis is, considered as the most economically important type of mastitis because of its higher prevalence and long term devastating effects as compared to clinical mastitis.

Production losses due to subclinical mastitis in Ethiopia cross breed dairy cows have been estimated to be 38 US$ per lactation per cow. Subclinical mastitis accounts for over 90% of the total loss in milk production [12]. However, most dairy farmers in the country normally do not recognize subclinical mastitis, which incidentally occurs at much higher frequency than clinical mastitis [11]. Information about the prevalence of the disease and factors associated with the disease as well as the pathogen involved is essential in designing prevention and control measures against the disease. Thus, this study was aimed to estimate the prevalence, to assess the risk factors and to isolate the major etiological agent of subclinical mastitis in the study area.

## Main text

### Materials and methods

#### The study area

Study was conducted in Bishoftu town which is found in the central high lands of Ethiopia at 47 km Southeast of Addis Ababa, the capital city of Ethiopia. The town is located at 8^o^45′ N longitude and 38^o^59′ E latitude at an altitude of 1880 m.a.s.l. It has an average annual rainfall of 1150 mm of which 84% falls down during the long raining season that extends from June to September, and the remaining during the short rainy season that extends from March to May. The mean annual minimum and maximum temperatures are 8.5  and 30.7 °C, respectively, and the mean relative humidity is 61.3% [13].

#### Study design, study animals and husbandry practice

A cross sectional study design was undertaken from November 2015 to March 2016 in selected 12 dairy farms found in Bishoftu town. The study animals were cross breed lactating dairy cows. There are 40 (small to large scale) dairy farms in Bishoftu town. Intensive management system is practiced in the dairy farms. Milking was performed two times a day by hand. There is no practice of mastitis screening and no dry period treatment; only clinical mastitis is treated. Hygienic practices such as udder washing, drying and post milking teat dip were practiced in the farms. Due to similarities in management practice variables like milking type, mastitis screening and udder and farm hygiene were not considered as explanatory variables. In this study daily milk yield (litres), body condition, age (years) and number of parity were the variables considered as risk factors (explanatory variables). Body condition was scored based on the guideline given by Nicholson and Butterworth [14].

#### Sampling method and sample size determination

The sample size was determined according to the formula given by Thrusfield [15]. It was calculated by taking 21.8% estimated prevalence from the previous report and 95% confidence level and 5% precision. Simple random sampling technique was employed.

$$ n = \frac{{z^{2} *P_{ \exp } (1 - P_{ \exp } )}}{{d^{2} }}, $$

where *n* =  required sample size, Z = the alpha value of 95% CI = 1.96, *P*_*exp*_  =  expected prevalence, *d*  =  desired absolute precision.

#### Milk sampling and screening

Milk samples were examined for visible abnormalities and screened using California mastitis test (CMT) according to the procedure given by Quinn et al. [16]. From each quarter of the udder, a squirt of milk sample was place in each of the cups on the CMT paddle and an equal amount of 3% CMT reagent (CMT, AHDB Dairy, UK) was added to each cup and mixed well. The reactions were graded as 0 for negative, + 1, + 2, + 3 for positive [16, 17]. Subclinical mastitis was diagnosed based on CMT results and the nature of coagulation and viscosity of the mixture (milk and CMT reagent), which show the presence and severity of the infection, respectively [18]. CMT positive samples were considered for bacteriological culture. For bacteriological culture, additional milk samples were collected from CMT positive quarters using sterile universal bottle. The sample was transported to the laboratory for further bacteriological test.

#### Bacteriological isolation and identification

CMT positive samples were bacteriologically examined according to the procedures given by Quinn et al. [16] and Sears et al. [19]. A loopful of milk sample collect from each infected quarter was inoculated onto MacConkey agar (HiMedia, India) and blood agar base (Oxoid, England) enriched with 7% defibrinated sheep blood. The inoculated plates were then incubated aerobically at 37 °C for 24–48 h. Staphylococci were identified based on catalase test, growth characteristics and sugars (mannitol and 1% maltose) fermentation on mannitol salt agar (Oxoid, England) and purple agar (Difco, France) and tube coagulase tests [16]. Identification of Streptococci were made based on Catalase test and its growth characteristics (hemolytic type and aesculin hydrolysis) on Edward’s media (Oxoid, England), CAMP test and its growth ability on MacConkey agar.

From Gram negative bacteria, *Escherichia coli* was the main focus and the isolation was based on their growth and lactose fermentation characteristics on MacConkey agar, its identification was made according to colonies reaction on the selective media EMB (Eosin Methylene blue) agar (Oxoid, England) and IMVIC (Indole, Methyl red, roges-proskaur, and citrate) test and additional tests like triple sugar iron (TSI) agar (HiMedia, India).

#### Data analysis

All the data collected were entered and stored in Microsoft excel and then imported to STATA Version 12 (Stata corp., college station, TX) for analysis. Pearson Chi square was used to evaluate the statistical significance of different risk factors with the result of CMT and bacteriology. Univariate and multivariable logistic regression analyses were performed to quantify crude and adjusted Odds ratio (OR), respectively. *P* value less than 5% (P < 0.05) was considered statistically significant. In cases of estimating the effect of different risk factors in terms of OR with corresponding 95% confidence interval, statistical significance was assumed if the confidence interval did not include one among its values. Descriptive analysis methods also used to indicate the prevalence of subclinical mastitis at cow and quarter level.

### Result and discussion

The CMT screening test indicates that 105/262 (40.1%) cows and 170/1048 (16.2%) quarters examined were positive for subclinical mastitis. From 105 CMT positive cows 101 (38.5%) cows and from 170 quarters 153 (14.6%) quarters were bacteriological positive.

In Ethiopia, subclinical mastitis is considered to be an important challenge for the dairy development. This study also indicates subclinical mastitis to be the major problem in the study area. Both the CMT and bacteriological result showed the prevalence of sub clinical mastitis in the study area to be very high. This result is in agreement with previous studies conducted in different parts of the country; Shirmeka [20], Mekebib et al. [21], Sori et al. [22], Workineh et al. [23] and Girma [24] who reported prevalence of 40, 34.8, 40.6, 38.2 and 34.4%, respectively. However, the prevalence of subclinical mastitis in this study is relatively higher than the report by Biffa et al. [25] and Lakew et al. [26] from Southern Ethiopia and South eastern Ethiopia, respectively. Subclinical mastitis is a complex disease and the prevalence could be affected due to variation in management system, age, milk yield, body condition, parity, environment and other conditions. Thus, these factors might have contributed to the observed differences in prevalence of subclinical mastitis.

In this study age, body condition score, milk yield, and parity were considered as a potential risk factors for the occurrence of subclinical mastitis. From the risk factors considered age and number of parity have statistically significant (P < 0.05) association with subclinical mastitis both in CMT and bacteriological examinations (Table 1). As showed in Table 2, the odd of being positive in age group ≥ 9 years was 5.7 times higher than the age group [2–6) years and the odds of being positive in age group [6–9) years was 2.25 times higher than the age group [2–6) years. In multivariable logistic regression there is difference between CMT result and bacteriology; it shows milk yield had significant effect on subclinical mastitis using bacteriological examination but not using CMT. The adjusted odd ratio in high milk producing cows was less than the adjusted odd ratio of low milk producing cows (Table 2). According to Erskine [27], primiparous cows have more effective defense mechanism than multiparous cow. The prevalence of subclinical mastitis is expected to increase when age increases as older cows have more exposure time to causative organisms of subclinical mastitis than young cows.

**Table 1:** Association of different factors with subclinical mastitis

Risk factors	Number of cows examined	CMT			Bacteriology		
		Number positive	Mean and 95% CI	p-value	Number positive	Mean and 95% CI	p-value
Body condition score							
Poor	7	3	42.9 (0–92.3)	0.92	3	42.1 (28.9–55.3)	0.78
Medium	198	78	39.4 (32.5–46.3)		74	37.4 (30.6–44.2)	
Good	57	24	42.1 (28.9–55.3)		24	42.1 (28.9–55.3)	
Milk yield (liters)							
≥ 15	106	39	36.8 (27.5–46.1)	0.37	36	33.9 (24.8–43.1)	0.21
< 15	156	66	42.3 (34.5–50.1)		65	41.7 (33.8–49.5)	
Age (years)							
[2–6)	163	51	31.3 (24.1–38.5)	0.001	49	30 (22.9–37.2)	0.001
[6–9)	81	41	50.6 (39.5–61.7)		39	48.1 (37.0–59.3)	
≥ 9	18	13	72.2 (49.3–95.1)		13	72.2 (49.3–95.1)	
Number of parity							
1–3	204	68	33.3 (26.8–39.8)	0.001	65	31.9 (25.4–38.3)	0.001
4–6	53	32	60.4 (46.8–73.9)		31	58.5 (44.8–72.2)	
≥ 7	5	5	100		5	100	

Keys: [for value included into the range,) for value not included into the range

**Table 2:** Multivariable logistic regression analysis of subclinical mastitis and bacteriology with various risk factors

Risk factors	Number of cows examined	CMT			Bacteriology		
		Number of positive	Crude odds ratio (95% CI)	Adjusted odd ratio (95% CI)	Number of positive	Crude odds ratio (95% CI)	Adjusted odd ratio (95% CI)
Body condition score							
Poor	7	3	1	1	3	1	1
Medium	198	77	0.86 (0.18–3.97)	0.74 (0.13–4.03)	74	0.79 (0.17–3.65)	0.70 (0.13–3.77)
Good	57	25	0.96 (0.19–4.73)	0.73 (0.12–4.37)	24	0.96 (0.19–4.73)	0.72 (0.12–4.26)
Milk yield (litres)							
≥ 15	106	39	1	1	36	1	1
< 15	156	66	1.25 (0.75–2.09)	2.06 (0.95–4.44)	65	1.38 (0.83–2.31)	2.32 (1.05–5.08)^a^
Age (years)							
[2–6)	163	51	1	1	49	1	1
[6–9)	81	41	2.25 (1.30–3.89)^a^	1.63 (0.76–3.53)	39	2.16 (1.24–3.74)^a^	1.48 (0.67–3.23)
≥ 9	18	13	5.70 (1.93–16.86)^a^	1.86 (0.39–8.73)	13	6.04 (2.04–17.8)^a^	1.79 (0.37–8.46)
Parity							
1–3	204	68	1	1	65	1	1
4–6	53	32	3.04 (1.63–5.68)^a^	2.34 (0.91–5.97)	31	3.01 (1.61–5.60)	2.39 (0.93–6.18)
≥ 7	5	5	–	–	5	–	–

^a^Significant associations

**Fig. 1:**
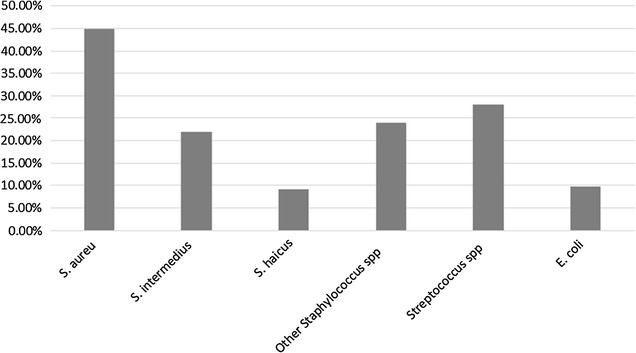
Bacterial isolates from milk samples collected from Bishoftu small, medium and small scale dairy farms

All 170 CMT positive milk samples at quarter level were examined and 153 (16.2%) were found culture positive. From the isolated bacteria 137/153 (89.5%) showed single growth, while 16/153 (10.5%) had mixed growth. The major pathogens isolated were *Staphylococcus aureus* (44.95%), *S. intermedius* (22%), *S. haicus* (*9.2%*)*, other Staphylococcus* spp. (23.9%), *Streptococcus* spp. (28.1%) and *E. coli 9.8%* (Fig. 1). The dominant isolate was *S. aureus* which is in agreement with the findings of Nesru et al. [28], Abdella [29] and Sori et al. [22].

In the study, *S. aureus* is the most prevalent organism isolated; this indicates that transmission occurs at the time of milking. The invariable hand milking practice and the absence of dry cow therapy among the dairy herds could lend a hand for sustained transmission of the contagious pathogens. *S. aureus* and other contagious microorganisms such as *Streptococcus agalactiae* are usually found in the teat canals, on teat or udder skin and also in infected udder [30, 31] and are the primary source of infection between infected and uninfected udder quarters and between infected and uninfected cows, usually during milking. Even though hygienic practices such as udder washing, drying, and post milking teat dip were practiced in the farms, these hygienic practices alone could not reduce the challenge of contagious mastitis pathogens poses since these pathogens especially *S. aureus* are widely prevalent. Thus, mastitis control measures such as testing cows by CMT, using gloves and changing gloves between cows or at least after milking subclinically or clinically infected cows and testing of new introduction into the herd should be practiced. Furthermore, due to low cure rate of *S. aureus* infections with antibiotic therapy during lactation, dry cow therapy and culling of chronically infected cows should be practiced. In mean time, the pre and post milking teat disinfection should be further strengthened to slow down the transmission of both contagious and environmental pathogens.

In conclusion, in Ethiopia, the subclinical form of mastitis received little attention and efforts have been concentrated on the treatment of clinical mastitis, however high economic losses often come from subclinical mastitis [32]. Ethiopian dairy farmers are not well informed about the invisible loss from subclinical mastitis and thereby there is no practice of subclinical mastitis screening tests. The present study showed a high prevalence of subclinical mastitis and Staphylococcus and Streptococcus species being the dominant bacterial causing subclinical mastitis. Thus, the above mentioned control strategy should be practiced to reduce the impact of the disease.

## Limitations of the study

Hygienic and other management practices were known to affect the prevalence of subclinical mastitis. In this study these important risk factors were not considered. Thus, failure to incorporate these factors should be considered as a shortcoming of the study.

## Abbreviations

BCS: body condition score
CMT: California mastitis test
*E*: *east*
EMB: eosin methylene blue agar
IMVIC: indole, methyl red, roges-proskaur, and citrate agar
OR: odds ratio
*N*: *north*
TSI: triple sugar iron agar
US$: United States Dollar
